# Adolescents Just Do Not Know What They Want: A Qualitative Study to Describe Obese Adolescents’ Experiences of Text Messaging to Support Behavior Change Maintenance Post Intervention

**DOI:** 10.2196/jmir.3113

**Published:** 2014-04-08

**Authors:** Kyla L Smith, Deborah A Kerr, Ashley A Fenner, Leon M Straker

**Affiliations:** ^1^School of Physiotherapy and Exercise ScienceFaculty of Health SciencesCurtin UniversityPerthAustralia; ^2^School of Public HealthFaculty of Health SciencesCurtin UniversityPerthAustralia; ^3^Health Psychology and Behavioural Medicine Research Group, School of Psychology and Speech PathologyFaculty of Health SciencesCurtin UniversityPerthAustralia

**Keywords:** telemedicine, text messaging, adolescent, obesity

## Abstract

**Background:**

Adolescents are considered a hard to reach group and novel approaches are needed to encourage good health. Text messaging interventions have been reported as acceptable to adolescents but there is little evidence regarding the use of text messages with overweight and obese adolescents to support engagement or behavior change after the conclusion of a healthy lifestyle program.

**Objective:**

The intent of this study was to explore the opinions of overweight adolescents and their parents regarding the use of text messages as a support during the maintenance period following an intervention.

**Methods:**

This paper reports on the findings from focus groups conducted with adolescents (n=12) and parents (n=13) who had completed an eight-week intensive intervention known as Curtin University’s Activity, Food and Attitudes Program (CAFAP). Focus groups were conducted three months post intensive intervention. Participants were asked about their experiences of the prior three-month maintenance phase during which adolescents had received tri-weekly text messages based on the self-determination theory and goal-setting theory. Participants were asked about the style and content of text messages used as well as how they used the text messages. Data were analyzed using content and thematic analyses.

**Results:**

Two clear themes emerged from the focus groups relating to (1) what adolescents liked or thought they wanted in a text message to support behavior change, and (2) how they experienced or responded to text messages. Within the “like/want” theme, there were five sub-themes relating to the overall tone of the text, frequency, timing, reference to long-term goals, and inclusion of practical tips. Within the “response to text” theme, there were four sub-themes describing a lack of motivation, barriers to change, feelings of shame, and perceived unfavorable comparison with other adolescents. What adolescents said they wanted in text messages often conflicted with their actual experiences. Parent reports provided a useful secondary view of adolescent experience.

**Conclusions:**

The conflicting views described in this study suggest that overweight and obese adolescents may not know or have the ability to articulate how they would best be supported with text messages during a healthy lifestyle maintenance phase. Further, supporting both engagement and behavior change simultaneously with text messaging may not be possible. Intervention texts should be personalized as much as possible and minimize feelings of guilt and shame in overweight and obese adolescents. Future research with text messaging for overweight and obese adolescents should incorporate clear intervention aims and evaluation methods specifically related to adolescent engagement or behavior change.

**Trial Registration:**

Australian New Zealand Clinical Trials Registry: ACTRN12611001187932; https://www.anzctr.org.au/Trial/Registration/TrialReview.aspx?ACTRN=12611001187932 (Archived by WebCite at http://www.webcitation.org/6LGSbk8d9).

## Introduction

The provision of effective health-related interventions for adolescents is a difficult task, particularly when traditional methods of communication are unlikely to be engaging [1]. Current mobile telephone usage trends show adolescents are increasingly using text messaging as a preferred method of communication [2]. Text messaging, also known as short message service (SMS), may therefore provide an acceptable means of delivering health messages in adolescent populations [3-6].

In adolescent weight management programs, text messaging shows promise as a feasible and acceptable method of communication [4,7]. Evidence from healthy weight adolescent groups support this concept of “acceptability” [8], yet it remains unknown whether text messaging is an effective means for fostering engagement or supporting behavior change.

Given the significant dropout rates reported in pediatric weight management programs [9] and lack of evidence for long-term weight maintenance in adolescence [10], there is much to be gained from developing an effective method for keeping participants engaged with a program. Text messaging may be a useful way of maintaining this engagement. De Niet et al [6] found that children and adolescents randomized to a text message treatment group were 3.5 times less likely than the control group to drop out of the maintenance period of a healthy lifestyle intervention but also showed that text message engagement declined over the 9-month period. Older pre-adolescent children (12 year olds sent 0.5 texts per week) were less likely to send responses compared with younger children (7 year olds sent 0.8 texts per week) [6]. This decline in adherence to monitoring over time has been documented previously [11], but adherence remains higher than other traditional methods of self-monitoring. Kornman et al [7] described only a modest level of adolescent engagement with their text messaging and email adjunct to the Loozit program, with adolescents responding to 22.0% of text messages over a 10-month maintenance period. The authors found that text messages asking for a response had more replies and text messages had a more immediate response than the emails. Whether text messaging can be used to maintain engagement of overweight adolescents in a healthy lifestyle program remains unclear.

Studies evaluating the effectiveness of text messages offer inconsistent support for the use of text messaging to support behavior change and/or weight maintenance [12-16]. Adolescents in the Loozit program rated the text message support during the 10-month maintenance period as “somewhat helpful”; however, the results suggested that the use of text messages developed for this intervention did not have a significant effect on primary outcomes at 12 or 24 months post program [7,15,16]. Other short-term studies assessing the effectiveness of text messaging interventions with adolescents suggest success only in those who had not previously engaged in the targeted behaviors before the intervention commenced [12] or who received additional intensive support (ie, intensive insulin therapy) in addition to text messages [13]. There remains a gap in the evidence regarding the effectiveness of text messaging interventions to support behavior change in overweight and obese adolescents.

There is also limited evidence regarding the best way to construct and send text messages for use with adolescents [17]. Factors to consider include the message tone, content and length, as well as the timing and frequency of text message delivery. Several studies have reported the wording and timing of delivered messages [3,6,7,12], but only two studies have considered the format of text messages to be sent regularly to support behavior change in adolescents [8] or obese adolescents [17]. Rigorous pilot testing of text messages has provided descriptions of what adolescents think they want [8,17], but whether adolescents actually do know and can articulate what they want in text messages and whether this is desirable for either engagement or intervention efficacy is unknown. There are currently no studies that have completed a detailed post-intervention evaluation of overweight and obese adolescent experiences of text messages, including perceptions of content, style, or usefulness. A lack of synchrony between what adolescents think they want and may actually like or find useful is suggested by evidence that overweight teenagers have indicated a strong preference for directive text messages, which seems to contradict their desire for autonomy [17].

Parents have not traditionally been involved in the development or refinement of text messaging interventions for adolescents. However, parents may play an important role in supporting adolescent engagement in research [1], providing healthful environments [18], modelling healthy behaviors [19], and supporting adolescent behavior change [20]. The potential for parents to offer valuable insight into adolescent experiences of text messaging interventions warrants exploration.

Current recommendations for research into text messaging interventions advise researchers to design their study with a strong theoretical foundation [21,22] and report on process evaluation after the intervention, to inform others about the best way to structure text messages, and effectively utilize this form of communication with adolescents [7,17,21,23]. Therefore, the aim of this study was to explore opinions of overweight and obese adolescents and their parents, who have participated in a multi-disciplinary healthy lifestyle program, regarding the use of text messages as a support during the maintenance period.

## Methods

### Study Design

This study relates to focus groups held during the maintenance phase of a multi-disciplinary healthy lifestyle program for overweight and obese adolescents aged 12-16 years and their parents. A wait-list controlled trial of Curtin University’s Activity, Food and Attitudes Program (CAFAP) has been conducted and is described elsewhere [24] along with its theoretical underpinnings [20]. Briefly, participants enrolled in CAFAP and were placed on a three-month waiting list to allow for a control period. Following this waiting list period, adolescents and parents completed the intensive phase of the program, involving twice-weekly group sessions for eight weeks. Sessions covered healthy eating, increasing physical activity, reducing sedentary behavior, and setting goals for healthy behavior change. Parents were also trained in behaviors around need satisfaction and goal setting to support their adolescent’s lifestyle changes [20]. The intensive phase was followed by a tapered maintenance phase over 12 months. The first three months of the maintenance phase were considered to be “high intensity” where adolescents received three text messages per week and one phone coaching session every two weeks. The next three-month period included a tapering of contact to “medium intensity”, where adolescents received a weekly text message and a monthly phone coaching session. The “low intensity” phase of the maintenance period occurred between six and 12 months post intensive program and included monthly text messages and quarterly phone coaching sessions. This study was approved by the Curtin University Human Research Ethics Committee (HR105/2011).

This qualitative study was performed three months after the eight-week intervention period of CAFAP, which coincided with the conclusion of the high intensity maintenance period. The intent of the study was to explore adolescents’ perceptions of text messages received, in combination with the observations and experiences of the parents, during the previous three months. Four focus groups were conducted with overweight and obese adolescents between September 2012 and April 2013 to cover the three waves of participants who completed the program at different times. Four focus groups were also completed by the parents of these adolescents. Focus groups were small (3-4) to encourage rich discussion of the text messages.

### Recruitment

Three months after completion of the intensive face-to-face CAFAP sessions, adolescents from each wave were invited by flyer, email, and text message to participate in a 60-minute focus group. Adolescents were offered a $30AUD gift voucher for participating in the focus group. Of the 35 adolescents invited, 16 agreed to participate and 12 attended the focus groups. Parents were recruited once their adolescent had agreed to participate in the focus group. All adolescents had a parent participate in the focus groups at the same time as they completed their own focus group.

### Text Message Development and Programming

CAFAP used an automated text messaging system to send predetermined but semi-tailored text messages to adolescents. During the first three months of the 12-month maintenance period, adolescents were sent a text message at 6pm on two weekdays and noon on one weekend day. The timing and frequency of text messages in this study was chosen as it reflected a midway point between previous adolescent trials [7,13] and incorporated evidence from associated formative research [25]. Adolescents chose the days that would best suit them at the conclusion of the intensive intervention phase. Two versions of the message plan were developed using the same text messages but in a different order to ensure that adolescents who were close friends would receive different messages. Texts were limited to 320 characters minus auto text (“Hi [first name], [text message], from the CAFAP Team”). Texts were sent to the adolescent’s personal phone in most instances, except where the adolescent did not have a personal phone or did not use it consistently. Some families requested the text also be sent to a parent’s phone to keep the parent involved. If text messages were not able to be delivered, research staff would follow up to manually send the text or contact the participant by phone if the text remained undelivered.

The contact during the maintenance period continued with the same theoretical base and key messages as during the intensive face-to-face contact period. Text contact was thus based on self-determination theory [26] and goal-setting theory [27] and focused on eating more fruit and vegetables, eating less junk food, being less sedentary, and being more physically active [20,24]. Message development also incorporated recommendations from current literature, by including a positive tone [6,17,23] and friendly but professional language [17]. Messages were constructed so as to be perceived as deriving from the research team [7,17] and were semi-tailored [28] by using first names and references to CAFAP or past text message contact [4-7,13,17,21]. The word “you” was frequently used to make facts relatable to adolescents [8] and a maximum of two reflective questions were included in each message [17]. Messages that provided options for behaviors were always framed to give adolescents a choice (eg, “You might like to…”) and recipes or testimonials from other teenagers (eg, “Other teens have found it helpful to…”) were used to enhance self-efficacy [17]. Suggestions for activities were based on affective beliefs to emphasize the enjoyment and social nature of participation [12] and reaffirmed the benefits of healthy eating and physical activity. Negative wording or the mention of triggers for unhealthy behaviors (eg, consumption of junk food) was avoided [17]. Adolescents were able to reply to text messages and receive a response, but were not expressly asked to reply. This approach was chosen to align with the theoretical underpinnings of text message development relating to fostering a sense of autonomy [20,26] and formative work suggesting adolescents may be reluctant to spend their own money on responses [25]. Text messages were designed to prompt behaviors and offer adolescents options for healthy behaviors, but they did not have to act on the texts if that was not their choice. The number of text messages sent to adolescents was measured on the SMS database. Five overall categories of texts were developed (general, goal setting, healthy eating, physical activity, and sedentary behavior) with messages tailored for weekday evenings and weekends. See Table 1 for examples of CAFAP text messages.

**Table 1:** Text message examples used to represent the content of text messages used in the intervention and typical adolescent responses from focus groups.

Category	Text message	Key strategies	Examples of adolescent responses^a^
Healthy eating	After dinner can be a good time to eat some fruit. If you had less than 2 bits of fruit today, you might like some tinned apricots & yoghurt for dessert?	Message including a ‘helpful tip’	*I liked it except I didn’t have any of that stuff.* [Adolescent 10 (A10)] *I’d prefer if you told me to have strawberries.* [A12]
			*It’s ok but I don’t really eat after dinner.* [A4]
Sedentary behavior	A CAFAP teen has found using an egg-timer is a good way of knowing it’s time for an active break after playing on the computer for 30 mins. You could choose to try it too.	Testimonial message	*Most probably wouldn’t influence my behavior because, I don’t know, I get addicted to my computer.* [A3]
Physical activity	What are the reasons you want to be more active? You might like to think about these when the going gets tough!	Intrinsic motivation, reflecting goal-setting process used during the program	*It makes you actually think about it. You have it there but you don’t actually think about it.* [A7]
Physical activity	How many steps have you done today? How about trying to do a few extra thousand steps this afternoon?	Reflective questioning	*There’s nothing wrong with it, but like saying a few extra thousand like is too much.* [A5] *It would make you think about doing more steps.* [A1]
Goal setting	Have a look at your goals for physical activity, sedentary behavior and healthy eating. Plan something fun to do this weekend to help meet your goals.	Bigger picture, reinforcing key CAFAP areas and goal setting.	*I have homework on the weekend, like every day… If I’m not doing homework I’m sleeping. Like pretty much.* [A11]
Healthy eating	How many bits of junk food have u had today? You could challenge yourself to see if you can go the rest of the day without any junk food. Some cut up fruit might be a sweet treat instead!	Reflective questioning and ‘helpful tip’	*I felt really guilty because I think I’d had ummm I’d had something, maybe a donut for breakfast.* [A2]

^a^Adolescents were assigned a code to distinguish between responders. The code ranges from A1-A12.

### Focus Group Protocol

#### Overview

Facilitators had completed formal training with a qualitative research expert covering focus group conduct prior to involvement in these focus groups and had prior experience in conducting focus groups with similar participants.

#### Adolescent Focus Groups

Adolescent focus groups were 60 minutes long and conducted by one author (KS). Each focus group was audiotaped with participant consent. In the adolescent groups, actual text messages were used to guide the discussion at the start of the focus group based on the work of Woolford et al [17] and previous difficulties noted by the research team when adolescents were asked to answer questions that required them to reflect on their experiences. Six messages were chosen from the database of texts sent during the high intensity maintenance phase to reflect the different content and key strategies used in the main text categories as outlined in Table 1.

The aim of using the six text messages was to explore the adolescents’ response to the message content. For each of the six messages, adolescents were asked:

- Did you like/dislike this message?
- What would you first think when you got this message?
- Is it a realistic message for you? (Is it *right* for you?)
- What’s good about it?
- What’s not so good about it?
- Did it make you think about changing your behavior (diet/activity/habit)?
- How could we make it better?

For the second part of the focus group, adolescents were asked about another six text messages (see Table 2) to understand their responses to the specific strategies used in the messages. The final part of the focus group included questions about general responses, timing, and how the text messages were used by adolescents.

**Table 2:** Text message examples used to represent the style of text messages sent in the high intensity maintenance period.

Question to adolescents	Example text message	Examples of adolescent responses^a^
What did you think of messages that asked you a question?	What was your sedentary behavior goal for today? Did you achieve your goal?	*For Monday to Fridays, it’s a bit hard because we have loads of double periods…and we’re sitting down for long periods of time so we’ve kinda destroyed our sedentary goal.* [Adolescent 12 (A12)]
What did you think of messages that reminded you about why being healthy was important?	Do you remember that the benefits of being more active include having a healthier heart and body chemistry, feeling less tired, sleeping better, being happier, and thinking better?	*Yeah. It helps you because it asks you a question but it instantly answers it for you.* [A4] *It’s good to remember. Feeling less tired would be good.* [A2]
What did you think of the ‘big picture’ messages?	Remember the key messages of CAFAP are: eat more fruit and veg, eat less junk food, be less inactive, and be more active.	*It actually like reminds you that you’re like on Earth and you’re sitting there playing video games…And I’ve gotta like do stuff and be proactive like normal people.* [A9]
What did you think of the messages that gave you healthy tips?	Think about how many veggies u had today. If u had less than your goal, try to add in 1 more piece tomorrow. How about some veggie sticks with one of the yummy dips u made at CAFAP?	*It was helpful. I often followed the tips.* [A8] *I found it good because I wouldn’t think of something like that.* [A6]
What did you think of the messages that included ideas from other teenagers?	Some teenagers have told us that cut up fruit salad for recess helps them reach their healthy eating goals. What about taking some tomorrow?	*These sound like healthy teenagers and sometimes I get a bit annoyed because I’m jealous. They have a better state of mind than me.* [A4]
What do you think about messages that are about what *not* to do?	Don’t eat junk food today or don’t spend the afternoon lying on the couch.^b^	*If you tell someone not to do something, they’re just going to do it, everyone knows that.* [A5]

^a^Adolescents were assigned a code to distinguish between responders. The code ranges from A1-A12.

^b^This text message was created to be tested in the focus groups based on current literature [17] but was not actually used in the intervention.

#### Parent Focus Groups

Parent groups were also 60 minutes long, conducted by one of two authors (LS or AF) and audiotaped with participant consent. This secondary view from parents is potentially helpful to attempt to validate the information provided by adolescents, particularly from interviews or focus groups where there is potential for participants to express a more socially acceptable view and obscure their true opinions. The group was asked three main questions regarding the experiences of their family in the previous three months, their opinions on the usefulness of the text message support, and any suggestions to better support adolescents after completion of the intensive eight-week program.

### Data Analysis

Following focus group discussions, responses to questions were transcribed verbatim by KS, with confidentiality ensured by coding of transcripts with subject identifier codes. The data was sorted and coded and assigned to categories based on similar phrases and topics. Coding was completed separately by two authors (KS and DK), with peer review checks by LS and AF to ensure the overall credibility of findings and interpretations [29]. Initial thematic analysis was based on the structure of the research questions [30] to identify theoretical constructs that described the experience of adolescents and parents. Themes were allowed to emerge using a mostly inductive approach [30]. Differences in interpretation were resolved by consensus. Underlying similarities and differences were evaluated and used to form the fundamental impression of the focus group discussions [31]. The data were triangulated with adolescent and parent interpretations compared [31] to give greater context to the data. By comparing and contrasting results from two groups with different viewpoints, we attempted to overcome the intrinsic biases associated with single group observations and explain the situation more fully [32]. Categories were amalgamated using Microsoft Excel and the major themes detailed using description and quotes from participants to support these findings [29].

## Results

### Demographics

Twelve adolescents with a mean BMI (body mass index) *z* score of 2.05 (SD 0.35) participated in the focus groups. The mean age of the adolescent participants was 14.3 (SD 1.5) years, with females overrepresented (92%, 11/12) when compared to all CAFAP participants who participated in the text message intervention (77% female, 33/43). A total of 13 parents, including 12 mothers and one father, participated in the parent focus groups. The majority of participants were white Australians from middle-low socioeconomic areas. Details regarding household characteristics were not further explored. The focus group participants included adolescents who had varying levels of success in adopting healthy behaviors at the three-month assessment and were likely a good representation of the overall group.

### Text Message Descriptive Statistics

In this intervention, 37% (16/43) of adolescents did not have access to their own phone regularly and had the text messages sent to their parent’s phone. These adolescents made up 33% (4/12) of the focus group participants. Of the parents, 60% (26/43) of parents in the intervention received a copy of the text messages, which included some parents of adolescents who had their own phone. A total of 2240 text messages were sent to adolescents (not including additional messages sent to parents only), eliciting 152 replies at a response rate of 6.79%.

### Participant Opinions

#### Overview

All attendees were generous with their feedback and engaged meaningfully in the discussions. Two distinct themes emerged from the adolescent and parent discussions relating to (1) what adolescents liked or thought they wanted in a text message to support behavior change, and (2) how they experienced or responded to text messages. Within the “like/want” theme, there were five sub-themes relating to the overall tone of the text, frequency, timing, reference to long-term goals, and inclusion of practical tips. Within the “response to text” theme, there were four sub-themes describing a lack of motivation, barriers to change, feelings of shame, and perceived unfavorable comparison with other adolescents. Themes are described and supported with quotes from adolescents (A) and parents (P) below.

#### More Casual and Personalized Text Messages Are Preferred

Adolescents were unanimous in their reaction to the tone of the texts, highlighting a need to make the messages less formal and more relatable to them. Parents expressed a similar view, indicating that the tone of the messages needed to be more personal.

More smiley faces. Smiley faces are good.

A1

They’re really proper. You need to abbreviate and stuff. Make it seem more human.

A3

They sound a bit rehearsed at times. A bit impersonal. Sometimes more casual…instead of Hi XX, it could be like Hey or Hey XX. More chatty. Because the ones that were being sent sound like for an adult. They’re too formal.

A5

Yes—make ‘em more 21st century.

A4

Make them custom for each person, because each person does a different amount of each thing…So for the stuff that we already do, the text messages should be like different for everyone.

A9

The SMS were too impersonal. It was like it was coming from a machine.

P7

#### Tri-Weekly Text Messages Were Too Frequent

Adolescents and parents all agreed that the messages were sent too frequently and as a result the reaction to the texts became increasingly negative over time.

If it had been say one a whole week, say on a Wednesday because that’s the middle of the week, to see how you’re going but to help as well, that’d be good. But three continuous in a week, that’s just like Shut Up!

A5

It’s just another form of nagging to them.

P7

They kept on coming. Like why can’t you let us do what we’re doing and then we tell you eventually when we come back.

A4

I know the messages got to a stage where, it’s almost like she would duck.

P10

With the messages, she was fine with them to begin with, but after that she only wanted to know about a few.

P6

Towards the end I would see CAFAP and put it to one side.

A2

#### Lack of Consistent Response on the Best Time to Receive Messages

Adolescent views varied regarding the best time to receive text messages, although most thought the timing needed to be specific for each individual.

7 o’clock in the morning would be better for me because it’s just before I go to school. It’s when you check your phone.

A8

No, no, no, no, no. If you text messaged me before school I would absolutely call you and go off at you because you ain’t texting me before school. I am a bad morning person and getting a text message from CAFAP would just blow it. It’s a teenager thing.

A5

Wednesdays are a good day.

A1

Fridays, so you get them before the weekend.

A8

Weekends are more helpful.

A2

Have more texts in the holidays.

A6

Don’t text me during school holidays because I will not get them. I go into hibernation.

A12

#### Adolescents Wanted To Be Reminded To Think About Their Reasons for Wanting To Change

The messages that included triggers for adolescents to think about their long-term goals, reasons for wanting to make changes, and their decision to participate in CAFAP were generally appreciated**.** The supportive nature of these texts seemed to be better received than text messages related to behavior change.

I mean cuz, it’s like where do you want to be from here, now? What are you going to do to get to that spot? And it just kind of motivates you to say “Oh, that’s my dream and I’m getting there.”

A10

Maybe like “Why did you choose to do this program? Remember those and keep going.” I like that.

A11

It reminds you that you’ve gotta do that because you’ve done the program but now you’ve got to do it yourself.

A12

#### Practical Tips Were Valuable Inclusions in Text Messages

A strong theme that emerged was the preference of adolescents to receive practical and relevant examples of behavior change. This approach of providing positive ideas for adolescents to choose to engage in seemed to be better received than the reflective questioning style used in the intervention or negative framed messages (what not to do) that were tested in the focus groups but not used in the intervention.

Having a healthy tip that you can actually do is good.

A1

The being happier and thinking better…sometimes I need an example. Like what am I going to do?

A2

#### Text Messages Were Not Effective Motivators for Change for Adolescents

Adolescents reported that although the text messages often acted as a reminder or an awareness raiser, they were not able to motivate behavior change. Adolescents were quick to come up with reasons for why the text message wasn’t applicable to them or why they wouldn’t be able to use the healthy tips. Adolescents appeared not to be interested in thinking too deeply about the text messages or having to adapt the content to suit their lives. Parents highlighted that adolescent lack of motivation was a common barrier and thought the text messages were not successful in encouraging behavior change.

That’s a good message because it tells you what you should do and should not do, but you’re still going to be inactive and you’re still going to eat junk food.

A4

Not really good. What’s wrong with it is (1) I don’t have an egg timer, (2) I don’t really plan on buying one, and (3) I don’t think that anyone would stay on the computer for [just] 30 minutes, I think they’d want to be on there for longer.

A5

She does talk about things like going to Zumba classes with a friend but it’s all talk, nothing’s been done.

P3

Don’t think it made any difference. But then it’s hard to tell what’s going on in there sometimes.

P12

She’s made all of these wonderful decisions; she’s at that point where she’s gotta keep motivated to stay there. And that’s where she’s having a little bit of trouble.

P9

#### Lack of Time and Tiredness Were Barriers for Adolescents to Participate in Healthy Behaviors

Adolescents repeatedly emphasized their lack of free time to plan for or perform healthy behaviors. They identified many cases where they disregarded text messages because they didn’t feel there was enough time to implement any of the strategies offered. Adolescents also reported being too tired to participate in healthy behaviors and this was supported by parent opinions.

I don’t have time; I don’t even eat breakfast in the morning. We just don’t have that much time.

A10

Homework. I didn’t have time to think about it [the text messages] at all. I’ve got like five projects at the moment.

A9

I answer it [the text] in my head but the thing is I’m already tired. So by the time you’re on your weekend, you just crash.

A2

But the exercise is not there. I think part of it is their school is really full on. They’re too tired.

P11

#### Adolescents Stated Some Texts Induced Feelings of Shame

Adolescents reported a sense of guilt or shame associated with messages that reminded them about healthy behaviors that they were not implementing. Parents also reiterated this sense of shame emerging for their adolescent, stemming from a number of texts.

Thanks for making me feel like crap, cuz I’m not the healthiest and I’m not the fittest and I feel really tired. And maybe I’m not as happy as I could be. [That] would just make me feel bad.

A3

(in response to the text: Do you remember that the benefits of being more active include having a healthier heart and body chemistry, feeling less tired, sleeping better , being happier, and thinking better?)

That just makes me feel really depressed if I’ve eaten a lot of junk that day.

A12

It’s good to have healthy reminder but I notice that my daughter does respond with a level of guilt or shame. It’s a reminder in a way that she’s not doing, she could be doing more.

P1

#### Hearing About What Other Teenagers Are Doing Was Not Motivating For Adolescents

Similarly, the majority of teens described their dislike for messages that included ideas or experiences from other teens, reiterating this sense of shame that others were improving their health while they were not.

I think “Oh, other teens are better than me? Like OK maybe I should catch up a bit because next time I see them they’ll all be skinny and I’ll be like…still…still here.”

A2

I think what my daughter rolled her eyes at the most was “the other teens” and she felt like she’s being compared again…she would just sort of turn off.

P4

A minority reported that they enjoyed hearing about how other teens were experiencing similar challenges; however, this was disparate to the perceptions of their parents’ reports, as well as the facilitators’ knowledge of the adolescents’ progress.

## Discussion

### Principal Findings

#### Overview

The present study contributes to the evidence base around experiences of overweight and obese adolescents and their parents in response to text messages designed to support behavior change in the maintenance period of a larger intervention. Adolescents described a sense of shame in response to the text message intervention, which was also observed by their parents, presenting a new issue for health researchers using text messaging with overweight and obese adolescents. In this study, overweight and obese adolescents’ stated preferences for text messages differed from how they actually responded to such text messages. This suggests that pre-intervention testing of messages may not adequately simulate what happens in “real life”. There may be differences between messages that adolescents say they like and those that are actually helpful in supporting behavior change. The consensus from adolescents was that the text messages were occasionally useful but sent too frequently and did not substantially help them to change their behaviors. Parents agreed that the text messages were too frequent and possibly too impersonal. They acknowledged there were some positive responses to the messages but the majority of the adolescents did not like to be reminded about healthy behaviors. These findings emphasize the uniqueness of overweight and obese adolescents and suggest a need to strengthen future intervention aims and evaluation methods.

#### Shame

Overweight and obese adolescents experience a sense of shame regarding their health and/or body [33,34]. The results of this study show that regular health-related text messages have the potential to heighten this sense of shame, which is a new issue for consideration by researchers using text messaging with overweight and obese adolescents. Based on the theoretical underpinnings of CAFAP [20], text messages were specifically worded to promote autonomy and support adolescent choices, yet many adolescents perceived the text messages as reminders of what they should be doing but weren’t doing. This response from overweight and obese adolescents differs to that described in similar studies, albeit shorter term, in adolescents of differing weight statuses [8] or diabetic adolescents [13] who reported to enjoy receiving health-related text messages. Similarly, suggestions about what other teens had found useful were not well-received as they had been in the pilot phase of a previous study [17], rather, these adolescents reported they disliked being compared to others. This response is potentially a significant barrier for overweight and obese adolescents to maintain healthy behaviors, with feelings of shame being related to poor mental health [34] and in turn to poorer self-efficacy and reduced ability to engage in lifestyle changes [35]. These feelings or circumstances may be unique to overweight and obese adolescents and suggest that results from trials involving non-obese adolescents may not be easily generalized to this group.

#### Want Versus Response

The results from this study suggest a notable difference between what overweight and obese adolescents say they prefer in a text message as opposed to what is actually useful to them in the maintenance phase following intervention. Adolescents reported that texts relating to their long-term goals were useful to motivate them, yet they had difficulty with maintaining behavior change and parents reported significant issues with adolescent motivation. Adolescents were able to point out flaws in the text message content and suggest improvements; however, many of these included strategies previously used or previously criticized by the adolescents themselves. Similarly, adolescents expressed a desire for practical tips to use in their daily lives reflecting current evidence [8,17], yet were quick to highlight reasons that would prevent them from regularly using the tips. They identified a number of barriers including lack of time or lack of resources (eg, particular foods not available), but weren’t able to suggest many ideas to overcome these. Interpretation of these results suggest that adolescents are able to identify idealistic concepts for text message style and content that may make theoretical sense, but have difficulty envisioning their actual response to such messages. This has potential implications for researchers doing pre-intervention testing with overweight and obese adolescents because the views they express may indicate that the intervention will be useful but might not be a true reflection of their experience in a text message intervention. Currently, the majority of the evidence around text message development for overweight and obese adolescents is based on the assumption that adolescents know what will be helpful to them in a text message and can articulate that; however, these results suggest that this may not be the case. Qualitative post-intervention research may provide the most contextual data regarding overweight and obese adolescents’ reaction to text messages, but even within these results there are inconsistencies between what adolescents say they want and what they actually respond well to.

#### Purpose of Text

Text messages are often used with adolescents to (1) encourage behavior change or maintenance, and (2) to foster engagement between adolescents and interventions; however, this research suggests that it may be difficult to concurrently achieve both of these aims in a weight-related intervention using text messages. The overweight and obese adolescents in this study did not like receiving text messages reminding them to maintain healthy behaviors, and thus such messages have the potential to damage the therapeutic relationship established during a face-to-face intervention and hence ongoing engagement.

In this study, overweight and obese adolescents’ responses to the text messages became increasingly negative over time. This suggests that their motivation may decline over time leading to failure to maintain behavior changes. Many parents in this study reiterated that adolescents didn’t like to be prompted about healthy things they could be doing, although they felt that it was sometimes useful to encourage behavior change. Despite adolescents reporting their dislike of the text messages, the healthy behavior focus may have had a positive effect on adolescents’ health. The effectiveness of using texts as healthy behavior prompts is not clear, with some evidence suggesting that this may be effective in promoting behavior change in the long-term [36,37], while other evidence suggests that eliciting a negative or shameful response to a message results in a reduction in self-efficacy and lack of behavior change [35]. This delicate balance between prompting behavior change and avoiding a shameful response may account for some of the differences reported in the literature relating to effectiveness of text messaging interventions in overweight and obese adolescents [4,17].

Conversely, messages not related to weight or healthy behaviors may be more effective at keeping adolescents engaged with an intervention. In this study, adolescents expressed a desire for more casual and positive text messages, which may have been more suited to preserving links with the intervention rather than eliciting behavior change. If the intervention aim was to preserve links with adolescents, then text messages would need to be carefully constructed to foster this sense of engagement but could be completely unrelated to the behavioral aims of the intervention. Message development based on self-determination theory would therefore aim to promote a sense of connection for the adolescent [26]. To our knowledge, the use of text messaging interventions to foster engagement has not been tested independent of behavior change and should be explored in future research.

The current findings suggest that future studies would benefit from generating clear aims for text messaging interventions specifically relating to either behavior change or engagement, and designing the texts with these aims in mind. Similarly, evaluation methods need to be strengthened and appropriate measures used to assess whether the text messages actually achieve their intended aims.

#### Timing

Adolescents expressed a number of differing opinions regarding the timing of the text messages, suggesting that it may be most acceptable if timing is personalized to each participant’s preferences. In the current intervention, messages were sent three times per week. Adolescents and parents consistently reported that the frequency of the text messages was excessive and they found that the messages became boring over time. This is in contrast to other studies with even greater frequencies of contact [4,5,8,12,13] where adolescents were reported to be accepting of daily texts, although 20% of participants in one study indicated that they got bored of receiving the same or similar text messages [13]. Interestingly, only one of these studies [4] was directed at overweight and obese adolescents, suggesting that this group may have unique perceptions regarding the frequency of supportive text messages and this may be related to how well they are achieving their lifestyle goals. Ngyuen et al [16] sent a text message once per month on average to overweight and obese adolescents but suggested that this dose was possibly too low to have an impact. The desire for less frequent text messages in this study may be related to the sense of guilt overweight and obese adolescents describe when they are reminded about changes they aren’t making, although it remains unclear as to whether this can prompt behavior change. These results highlight how messages can possibly invoke an undesirable response in overweight and obese adolescents. Text messages become personal because they are sent to the participant’s phone and differ from other health messages on media such as television where the individual may be able to more easily ignore a message as they are perceived as not relevant to them. When an individual receives and reads the text message, they will make a decision as to how relevant or useful the information is for them. From the responses obtained, it appears that, in overweight and obese adolescents, health messages may invoke a response that reduces their motivation to change rather than stimulating them to continue to take action to increase their physical activity or improve their diet. Health researchers may therefore need to modify the logistics of text message delivery to best suit the aims of their intervention, be that engagement or behavior change.

#### Style

Focus group participants were unanimous in their call for more casually worded text messages. Adolescents have previously expressed a preference for health care providers to communicate via text using a natural tone while avoiding the use of text message “slang” as frequently used in adolescent-to-adolescent contact [17]. Despite attempts to use less formal language than in the previous study, our research suggests that the tone we used was perceived as too professional by adolescents. The addition of symbols and emoticons (eg, exclamation marks and smiley faces) to text messages may help to convey a less formal tone. Although most text messaging studies have used tailored messages [4-7,13,17,21,28], our research suggests that overweight and obese adolescents want them to be further customized to their individual goals and experiences. Adolescents also reported that negatively worded texts (eg, “don’t eat junk food”) were likely to increase the likelihood of actually performing those behaviors, supporting recent findings by Woolford et al [17]. These authors [17] suggested that overweight and obese adolescents often want to avoid the psychological work associated with reflection and instead prefer tips and testimonials from other adolescents, yet our results suggest that overweight and obese adolescents in actuality do not find these helpful to assist with behavior change.

#### Parents

No other studies to our knowledge have included parent perceptions of their overweight and obese adolescent’s responses to receiving supportive text messages. Including parent views helps to understand the differences in opinions that adolescents might express to parents when at home, as opposed to researchers. This triangulation of the data provides a richer sense of what the “real” truth may be. The views expressed by parents in this study were generally aligned with the opinions described by adolescents and were also useful in identifying inconsistencies described by individual adolescents whose views consistently differed from the overall group.

### Limitations

As with all qualitative studies, there are potential limitations associated with participants reporting a view that may be perceived as socially acceptable, rather than their true opinion. It is possible that overweight and obese adolescents in particular may be prone to report in this way. The focus group facilitators in this study were known to the participants, which may have influenced participants to offer a more positive view on the use of text messages; however, the sometimes negative opinions expressed by parents and adolescents suggest this was not the case. The small size of the focus groups may have either encouraged or impeded discussion. It is also not possible to determine directly how the messages affected their behavior and if they took action. For example, some irritation with the messages may still have served as a reminder to increase their physical activity or improve their diet. The strengths of this study include an appropriate qualitative design to address the research question, a range of informants with directly relevant opinions for the research question, the inclusion of parents and adolescents, and provision of novel, in-depth information about the challenging topic of providing support to overweight and obese adolescents following an intensive intervention period.

### Implications for Research and Practice

This study presents several implications to consider in future research. First, overweight and obese adolescents may not know or have the ability to articulate how they would best be supported during the maintenance phase. Second, researchers should generate clear aims when planning a text message intervention, and develop appropriate methods of measuring success relative to the specific aims (for example engagement or behavior change). Third, timing and content of messages may need to be more individualized and may require ongoing input from adolescents to understand their preferences for receiving text messages. Fourth, reports of parent experiences may offer a useful secondary view of adolescent responses to text message interventions. Last, overweight and obese adolescents might be more susceptible to feelings of guilt or shame than non-obese adolescents when reminded about maintaining behavior change.

### Conclusions

Findings from this study suggest that text messages may have a useful role in interventions for overweight and obese adolescents but that adolescent opinions pre-intervention may not be very predictive of their actual experience and that it may be difficult to achieve both enhanced engagement and behavior change simultaneously. The results emphasize the importance of message tone, content, and timing as these factors appear to impact on how the messages will be received by overweight and obese adolescents. The potential to initiate feelings of shame in adolescent recipients of text messaging was identified as an important issue. The study also supported the use of process evaluation to help inform further interventions, and highlights the value of parent and adolescent reports. Future research should be clear on the engagement or efficacy aims for an intervention, develop messages to accommodate adolescent preferences in conjunction with an appropriate theory, and employ appropriate evaluation measures of engagement or effectiveness.

## Abbreviations

AUD: Australian dollars
BMI: body mass index
CAFAP: Curtin University’s Activity, Food and Attitudes Program
SMS: short message service
